# Examining the significance of fingerprint-based classifiers

**DOI:** 10.1186/1471-2105-9-545

**Published:** 2008-12-17

**Authors:** Brian T Luke, Jack R Collins

**Affiliations:** 1 Advanced Biomedical Computing Center, Advanced Technology Program, SAIC-Frederick, Inc., NCI-Frederick, Frederick, MD 21702, USA

## Abstract

**Background:**

Experimental examinations of biofluids to measure concentrations of proteins or their fragments or metabolites are being explored as a means of early disease detection, distinguishing diseases with similar symptoms, and drug treatment efficacy. Many studies have produced classifiers with a high sensitivity and specificity, and it has been argued that accurate results necessarily imply some underlying biology-based features in the classifier. The simplest test of this conjecture is to examine datasets designed to contain no information with classifiers used in many published studies.

**Results:**

The classification accuracy of two fingerprint-based classifiers, a decision tree (DT) algorithm and a medoid classification algorithm (MCA), are examined. These methods are used to examine 30 artificial datasets that contain random concentration levels for 300 biomolecules. Each dataset contains between 30 and 300 Cases and Controls, and since the 300 observed concentrations are randomly generated, these datasets are constructed to contain no biological information. A modest search of decision trees containing at most seven decision nodes finds a large number of unique decision trees with an average sensitivity and specificity above 85% for datasets containing 60 Cases and 60 Controls or less, and for datasets with 90 Cases and 90 Controls many DTs have an average sensitivity and specificity above 80%. For even the largest dataset (300 Cases and 300 Controls) the MCA procedure finds several unique classifiers that have an average sensitivity and specificity above 88% using only six or seven features.

**Conclusion:**

While it has been argued that accurate classification results must imply some biological basis for the separation of Cases from Controls, our results show that this is not necessarily true. The DT and MCA classifiers are sufficiently flexible and can produce good results from datasets that are specifically constructed to contain no information. This means that a chance fitting to the data is possible. All datasets used in this investigation are available on the web.

This work is funded by NCI Contract N01-CO-12400.

## Background

It is well established that early detection of cancer often results in a better prognosis. This detection has relied on measuring the concentration of a particular protein or biomarker, such as cancer antigen (CA)-125 for ovarian cancer and prostate specific antigen (PSA) for prostate cancer. Unfortunately, many of the commonly used biomarkers have a low sensitivity and/or specificity which necessitate the search for new biomarkers. Clinically it is useful if the measurement of a biomarker be obtained from a readily available biofluid, such as blood, urine, tears, or mucous. Bioinformatic analysis of data obtained from biofluids may result in identifying new biomarkers.

The standard procedure is to obtain biofluid samples from individuals with known histologies and perform an search of experimentally measured quantities, or features, to construct and test a classifier. This is done by dividing those individuals with and without a given disease into a training set and a testing set. The training set is used to construct a classifier from a subset of the features such that it accurately determines whether an individual has the disease. If such a classifier can be found, the testing samples are then examined to verify its accuracy. The goal of this procedure is to construct a classifier that can effectively be used on the underlying population; which Ransohoff denoted as generalizability [1,2].

While multiple biomarkers can classify a given individual better than a single biomarker [3], and it has been argued that tens to hundreds of biomarkers may be required [4], it is important to examine the way in which these markers are used in a classifier. While many forms of a classifier are possible, any classifier can be considered to lie between two possible extremes. At one extreme are classifiers denoted fingerprint-based classifiers, and at the other are classifiers denoted biomarker-based classifiers.

As the name implies, a fingerprint-based classifier is similar to the forensic procedure that determines whether or not a given individual was at a particular location. It uses a subset of the available features, or panel of markers, to construct a pattern and this overall pattern, or proteomic fingerprint, is used to identify the closest matching individual. In disease classification, if the match to an individual with a known histology (diseased or healthy) is sufficiently close, then the tested sample belongs to an individual with the same histology.

An example of a fingerprint-based classifier is the medoid classification algorithm (MCA) used in many studies from the laboratories of Emmanuel Petricoin and Lance Liotta [5-10]. This procedure scales the set of N selected feature values such that each training sample represents a point in an N-dimensional unit hypercube. A test sample is then scaled and placed in this hypercube, and if it is sufficiently close to one of the training samples it is given the same histology as this training sample. Every sample in the testing set must have a sufficiently similar sample in the training set, or else a prediction cannot be made.

The other extreme for classifiers is represented by a standard biomarker-based classifier. Here filtering methods are used to determine if the values of an isolated feature sufficiently distinguishes between diseased and healthy individuals. If a small number of such features are found, and their predictive ability is not caused by a bias in the study design, these features represent putative biomarkers and the classifier only uses these features. In a study of individuals with and without colorectal cancer it was found that the blood concentration of the complement C3a-desArg is elevated in individuals with either colorectal polyps or colorectal cancer [11,12]. Other studies have shown that complement C3a-desArg is also elevated in individuals with benign prostate hyperplasia [13,14] and Type-2 diabetes [15]. Therefore, a sufficiently low concentration of complement C3a-desArg in the blood may be sufficient to exclude any of these conditions; extra tests would have to be performed on an individual with a high blood concentration to correctly identify the condition.

**Figure 1:**
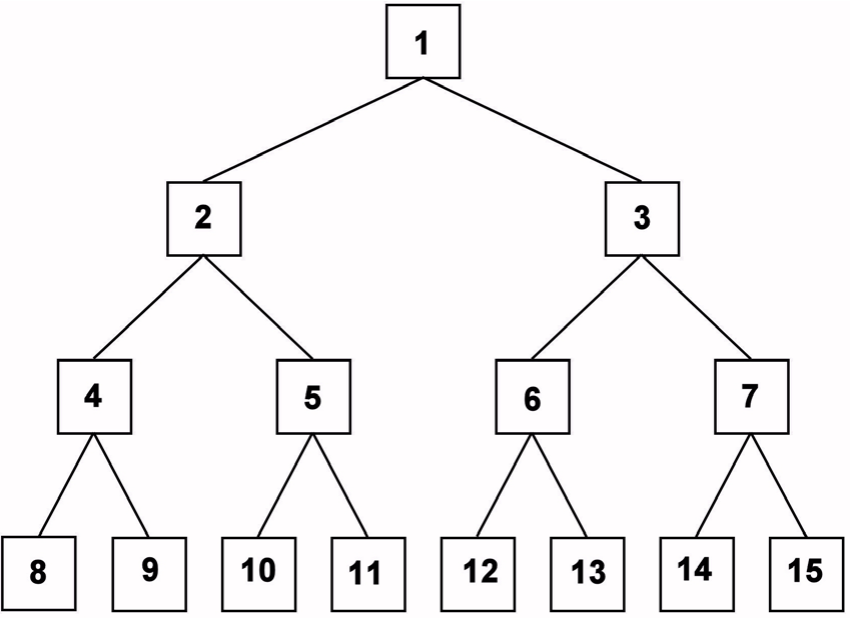
**Node numbering for the decision trees**. This investigation uses a decision tree containing seven decision nodes (Nodes 1 through 7) and eight terminal nodes (Nodes 8 through 15).

A decision tree (DT) classifier [16-28] can be considered to be between these extremes. If a sufficiently accurate DT classifier only requires a single decision node, then the feature used by this node represents a putative biomarker and this is a biomarker-based classifier. Since only the initial, or root node acts on all samples in the training set, any additional nodes only examine a subset of the training samples, and the members of this subset depends on the features used in any preceding nodes. The decision tree shown in Figure 1 contains seven decision nodes (labeled 1 through 7) that produce eight terminal nodes (labeled 8 through 15). If a feature used in decision node 2 is changed to another feature, for example, then the samples that are passed to nodes 4 and 5 would be changed. This would affect the quality of the discriminators for these latter nodes and the quality of the classifier. In other words, the optimal features to use in nodes 4 and 5 depend upon which features are used in nodes 1 and 2, while the optimal features in nodes 6 and 7 depend upon what features are used in nodes 1 and 3.

The main point is that a fingerprint-based classifier depends on the pattern of feature values across all N features used in the classifier. Changing one of the features used in an MCA classifier would necessarily change the location of all training samples in the N-dimensional unit hypercube and may drastically alter the classification accuracy for the testing samples. A multi-node DT has some of this property in that changing the features used in Nodes 1 through 3 in Figure 1 will change some or all of the sample subsets passed to decision nodes 4 through 7, and therefore change the classification accuracy and the optimal features to use in these latter nodes.

It is understood that if bias can be removed from consideration, a single feature that correctly distinguishes healthy from diseased individuals represents a putative biomarker that may be directly involved with the disease progression or with the host's response to this disease. Gillette and coworkers [29] have argued that the proteomic pattern or fingerprint associated with a panel of markers can be thought of as a single biomarker. Therefore, it has been stated [30] that if a fingerprint-based classifier is able to sufficiently predict the histology of individuals in an independent testing set, then this classifier must reflect some underlying biological principles. This assumption of accurate predictions being a necessary and sufficient condition for biological significance is tested in this manuscript. If any given classifier is able to accurately classify both a training set and a testing set using a panel of markers from a dataset that contains no biological information, then this association between good results being a necessary and sufficient condition for an underlying biological principle is disproved.

A random number generator is used to construct 30 datasets that contain no biological information. As described in the Methods section, each dataset contains the same number of Cases and Controls and each sample contains random values for 300 features. Current microarray and mass spectroscopic studies generate far more than 300 features, so this study investigates the flexibility of a classification algorithm instead of exploring the "curse of dimensionality." The number of Cases and Controls were set to 30, 42, 60, 90, 150, and 300; and five random datasets were constructed for each number of Cases and Controls. Therefore the smallest five datasets contained random feature intensities for 30 Cases and 30 Controls, and they represent situations where a chance fitting [1,2] of the data may be possible. The largest five datasets contain 300 features and 600 samples (300 Cases and 300 Controls) and a chance fitting of the data is not expected. All 30 datasets are available on the web [31] and any algorithm that produces good classification results can only do so by chance. Any acceptable classification disproves the sufficiency condition between accurate classification results and biological information. In other words, this "proof by counter example" argues against the contention that an accurate classification is sufficient to assume a biological relevance, and may underline the disconnect between many accurate classification studies and the lack of biomarkers that have been approved by the FDA.

In this study, the DT and MCA methods are used to examine these 30 different datasets. The DT procedure uses the symmetric decision tree shown in Figure 1 with seven decision nodes and eight terminal nodes, though for some runs pruning is performed for a putative classifier prior to determining its classification accuracy. The MCA method is used to construct classifiers containing five, six, or seven features from the set of 300. A complete analysis of these datasets would require an exhaustive testing of all possible sets of seven features in all possible orders for the DT method and all possible sets of five, six and seven features for the MCA method. Since this is not computationally feasible, a modified Evolutionary Programming (EP) algorithm [32] is used to search for near-optimal sets of features. This procedure selects sets of features that are passed to the DT and MCA algorithms to construct putative classifiers. This EP algorithm uses the classification accuracy of the putative classifiers to construct a final population of classifiers that accurately predict the histology of the samples.

Since the EP procedure is a stochastic search algorithm that samples a small subset of the available sets of features, finding the best set of features in a given run is not guaranteed. Therefore, for each classification method and dataset, multiple runs are performed. The DT procedure is run four times for each dataset, each with a different seed to the random number generator. In two of the runs no pruning is performed and in the other two a decision node is converted to a terminal node of it contains less than 4% of either the Cases or Controls. Each dataset is also examined twice by the MCA method for each number of features (five, six or seven). These two runs not only use different seeds to the random number generator but use a different ordering of the samples since (as described in the Methods section) the final result depends upon this ordering. Since finding the best set of features is not guaranteed, the results presented here should be taken as a lower bound, or minimum estimate, of the sensitivity and specificity that would be obtained for each procedure if an exhaustive search were performed.

## Results and discussion

A summary of the classification results for these artificial datasets is shown in Table 1. The first column lists the number of Cases and Controls and the rows correspond to the best results obtained from the five corresponding datasets. The first section of results in Table 1 lists the highest quality (sensitivity plus specificity as percentages) for the DT algorithm. Since each dataset was examined four times, the qualities represent the best results over 20 runs (four runs using five different datasets). The DT classification accuracy for the best and 200^th ^best classifier for each of the four runs using each dataset is listed in Additional file 1. The second section of results in Table 1 lists the highest quality (sensitivity plus specificity) for the MCA classifier using five, six or seven features. Since each dataset was examined twice for a given number of features, these results represent the highest quality obtained over 10 runs (two examinations of five datasets). The MCA qualities for the best and 200^th ^best classifier in each of the six runs for each dataset are listed in Additional file 2.

**Table 1:** Highest quality obtained from the DT and MCA classifiers

**Cases & Controls**		**MCA**		
	**DT**	**5 Peaks**	**6 Peaks**	**7 Peaks**
30	200.0	200.0	200.0	200.0
42	190.5	197.6	197.6	197.6
60	178.3	193.3	193.3	195.0
90	166.7	187.8	188.9	191.1
150	155.3	183.3	185.3	187.3
300	138.3	170.3	179.0	180.3

The reported quality is the largest sum of the sensitivity and specificity (in percent) found across the five random datasets for each number of Cases and Controls for a decision tree (DT) using at most seven decision nodes and the medoid classifier algorithm (MCA).

It should be noted that, for the MCA algorithm, each time the Cases were examined before the Controls all 200 classifiers produced a sensitivity of 100%, while each time the Controls were examined before the Cases the specificity was always 100%, independent of the number of Cases and Controls. This is a design feature of the MCA algorithm. Each time a sample is examined it is either placed in an existing cell or it becomes the medoid of a new cell. If only Cases are initially examined, they have to be placed in an existing Case-cell or create a new Case-cell. Either situation produces a correct classification of this Case sample. Though the exact sensitivity and specificity depend upon the order of the samples examined, their sum is relatively constant for the different ordering (see Additional file 2).

The DT classifier shows that the accuracy of the best identified classifier decreases as the number of samples increases. All of the 20 runs for the smallest datasets (30 Cases and 30 Controls) identified at least one decision tree whose average sensitivity and specificity was 95% or better and three of the 20 runs found at least one decision tree that produced perfect results (sensitivity = specificity = 100%). In fact one of these runs identified at least 200 unique decision trees that yield perfect results (see Additional file 1).

For the datasets with 60 Cases and 60 Controls, the runs identified at least one decision tree whose average sensitivity and specificity ranged from 85% to over 89%. The overall results for the best decision tree and a hypothetical division into a training set and a testing set is shown in Table 2. The training set has a sensitivity and specificity of 95.0 and 85.0%, respectively, while the testing set has a sensitivity of 90.0% and a specificity of 85.0%. It should be stressed that this division is not the only one that places 40 Cases and 40 Controls in the training set and 20 Cases and 20 Controls in the testing set while preserving the character of the eight terminal nodes as Case-nodes or Control-nodes: there are approximately 1.09 × 10^27 ^unique ways that these 120 samples can be placed into this specific division.

**Table 2:** Hypothetical placement of 60 Cases and Controls in training and testing sets

	**Node 8**	**Node 9**	**Node 10**	**Node 11**	**Node 12**	**Node 13**	**Node 14**	**Node 15**
**Overall**	0:6	15:0	0:17	2:0	35:9	2:8	2:20	4:0
**Training**	0:5	10:0	0:11	2:0	23:6	1:5	1:13	3:0
**Testing**	0:1	5:0	0:6	0:0	12:3	1:3	1:7	1:0

Overall placement of 60 Cases and 60 Controls (Cases:Controls) in the eight terminal nodes of the best decision tree shown in Table 1 and a hypothetical distribution between a training set and a testing set. Features 198, 140, 99, 68, 41, 95, and 251 are used in decision nodes 1 through 7 (Figure 1), respectively.

For the datasets with 90, 150, and 300 Cases and Controls, at least one run identified a decision tree with an average sensitivity and specificity above 83.3, 77.6, and 69.1%, respectively. The best results presented in Table 1 should be taken as lower bounds to the accuracy for a random dataset containing no biological information due to the small population size and number of generations in the modified evolutionary programming (mEP) search [32] and a modest search for the optimum cut points for each decision tree.

Significantly better results are obtained when the MCA method is used to fit the random datasets. If only five features are used, which is the minimum number considered in many previous publications [5-10], all 10 runs found at least one classifier that produced perfect results (sensitivity = specificity = 100%) for the datasets with 30 Cases and 30 Controls (see Additional file 2). When six or seven features are used, all 10 runs again found at least one perfect classifier, with two of the six-feature runs and four of the seven-feature runs producing final populations with at least 200 perfect classifiers. When the number of Cases and Controls is increased to 42, the best results yielded an average sensitivity and specificity of 98.8%, independent of the number of features. For 60 Cases and 60 Controls, both the five-feature and six-feature runs found at least one classifier with an average sensitivity and specificity of over 96.6%, while the seven-feature runs found at least one classifier with an average sensitivity and specificity of 97.5%.

As described in Methods, the MCA classifier is constrained so that at most two-thirds of the Cases and Controls are used to establish Case and Control proteomic fingerprint patterns, respectively. This means that at least one-third of all Cases and Controls are not needed to establish these fingerprints and can represent a testing set.

Table 3 lists the results for two different MCA classifiers using the same dataset treated by the DT classifier in Table 2. The MCA results in Table 1 and Additional file 2 first examined all Cases or Controls and then all samples in the other group, but for the results in Table 3 the samples were reordered so that there was a intermixing of the 60 Cases and 60 Controls. Both classifiers effectively used 40 Cases and Controls for the training set and 20 Cases and Controls for the testing set. They have a sensitivity and specificity of 97.5% for the training set and 95.0% for the testing set. Though all samples used to define a medoid must be part of the training set, there are still 1.51 × 10^9 ^unique ways that the remaining samples can be divided between training and testing sets for the first classifier and 9.49 × 10^6 ^unique ways to divide the remaining samples for the second classifier to obtain the division shown in Table 3. These two results were obtained using different seeds to the random number generator in the mEP search, and additional classifiers could be determined using a different seed or a different ordering of the samples in the dataset.

**Table 3:** Two medoid classification results for the same dataset

	**Training Set**						**Testing Set**			
	**Cases**			**Controls**			**Cases**		**Controls**	
**Result**	**Medoid**	**Corr**	**Incorr**	**Medoid**	**Corr**	**Incorr**	**Corr**	**Incorr**	**Corr**	**Incorr**
**1**	35	4	1	34	5	1	19	1	19	1
**2**	36	3	1	36	3	1	19	1	19	1

These results are for the same dataset as in Table 2 after a single mixing of the order of Cases and Controls after a hypothetical division of the samples into a training set and a testing set. The first classifier uses features 79, 114, 135, 137, 140, 224 and 300, and the second uses features 62, 65, 141, 146, 156, 211, and 229.

For the datasets with 90, 150, and 300 Cases and Controls, the MCA results show a monotonic decrease in the average sensitivity and specificity as the number of samples increases. For the largest dataset (300 Cases, 300 Controls, and 300 features), at least one seven-feature classifier produced an average sensitivity and specificity above 90.1%.

It has been argued that good classification results for a test set that in no way is used to determine the classifier necessarily implies that the classifier is based on some underlying biological information [30]. The results presented here show that good classification accuracy is not a sufficient condition to imply a biological basis for studies that use a DT or MCA classifier. If a good classification result implies a sensitivity and specificity of at least 85%, a decision tree with at most seven decision nodes can obtain this result from a random dataset containing more than 60 Cases and 60 Controls. If this threshold is increased to 90%, a decision tree can achieve this accuracy for a random dataset containing fewer than 60 Cases and 60 Controls, while a medoid classification algorithm achieves this accuracy with a pattern of seven features for a dataset containing 300 Cases and 300 Controls.

These results show that the quality of these classifiers will not decrease if more features are used in the fingerprint. Increasing the number of features into the hundreds [4] assures that a dataset containing even greater numbers of samples can be fit by chance [1,2] using a fingerprint-based classifier. It is important to note that these results are obtained for datasets containing only 300 features for each sample. Current separation technologies which yield multiple mass spectra for each sample as well as microarray studies produce many times more features per sample than considered here. Increasing the number of available features for each sample will also increase the quality of the classification using a DT or MCA, whether or not the dataset contains any biological information.

In a response to criticism that different studies used different features to accurately classify individuals with a given disease [33], it was stated that "the generation of multiple combinations of diagnostic features from the same starting data is a logical consequence of the complexity of the information content" [34]. The results in Tables 2 and 3 demonstrate that this statement is not absolutely true. Three classifiers each used seven features to accurately classify the same dataset that contains no biological information and only feature 140 was used in both the DT and an MCA classifier. The second MCA classifier used a set of seven features that were completely different from the 13 used in the other two. This is not a result of the complexity of the information content of the dataset, since it is designed to contain no information, but is due to the flexibility of the classifiers and their ability to generate a good fit only using noise.

It should be stressed that this investigation only examines the classification accuracy of fingerprint-based classifiers. In a fingerprint-based classifier, different combinations of features are examined and the "panel of markers" that produces the best result can be considered a single biomarker [29]. There is no point in examining each feature in this panel, since it is their concerted action that produces the classifier, and identifying the specific protein responsible for each of these peaks [35] would not be sufficient to claim that they represent biomarkers. Pre-screening potential features for their discriminating ability before using them in the final classifier [36] is representative of a biomarker-based classifier and is outside the scope of this investigation. In a fingerprint classifier, the proteomic pattern obtained from the panel of markers is what determines whether or not the individual has a given disease independent of the discriminating ability of individual features within the panel. For example, Zhang and coworkers used a panel of seven features in a decision tree classifier to diagnose patients with diffuse large B-cell lymphomas (DLBCL) [37], and none of these features showed significant differences between individuals with and without DLBCL. The MCA procedure [5-10] is an example of a pure fingerprint-based classifier. In this investigation, the decision tree classifier was also cast as a fingerprint-based method since no metric was used to determine which feature or cut point would be used at a particular decision node. Even if a metric such at the Gini Index or information gain were used, the final decision tree would still have some fingerprint qualities in that the feature selected for a given decision node is highly dependent upon which feature was selected for the preceding decision node. The more concerted the action of the features becomes, the more the classifier becomes fingerprint-based.

The final point is that the results presented here can be considered a chance fitting of the data [1,2], but the Additional files 1 and 2 show that there is no luck involved. For datasets with 300 features and 60 Cases and 60 Controls, the DT classifier was able to find several different classifiers with an average sensitivity and specificity of above 85% for each dataset. At least 200 unique 7-feature MCA classifiers produced an average sensitivity and specificity above 90% for each of the five datasets with 90 Cases and 90 Controls. The good classification results are simply due to the mathematical flexibility of the classifier.

## Conclusion

A previous publication has shown that a very accurate fingerprint-based classifier constructed from a finite number of samples is not necessarily generalizable to the underlying population [14]. This report extends these results to show that the high accuracy of a fingerprint-based classifier does not necessarily imply any underlying biological information since accurate results are obtained for a decision tree and a medoid based classifier using random datasets with no biological information. A classifier that correctly fits the data is a necessary condition to reveal biological relationships, but it is not sufficient.

It has been argued that the measured change in classification accuracy for a dataset and the same dataset with the class labels (histologies) permuted may be a way to measure the significance of the original classification [36]. Though this will be examined in detail in a later publication, preliminary results suggest that the drop in classification accuracy for the permuted dataset may be exaggerated if a filtering method is used to identify putative biomarkers prior to constructing the final classifier and the original dataset contained a putative biomarker. Therefore, comparing the classification accuracy for a given dataset against the accuracy of a comparably sized dataset containing random features (i.e. no biological information) may be a better test.

All 30 random datasets are available online [31] so that other classification algorithms can be examined. Included with the datasets is information that more thoroughly describe the DT and MCA results. In addition, a more extensive description of the DT and MCA algorithms used here as well as the actual programs is available [38].

## Methods

Since all 30 datasets have 300 peak intensities, the first step is to set a maximum intensity for each peak. The maximum intensity for each peak is set to a random number between 0.0 and 200.0. For example, Peak 64 is allowed to have a maximum intensity of only 1.055, while Peak 131 has a maximum intensity of 197.9. A random number in the range (0.0,1.0) is multiplited by the maximum allowed intensity to obtain the intensity for a feature in a given sample. A different seed to the random number generator is used for each dataset so that the first sample, for example, has a different intensity for each peak in each dataset. Since the average maximum intensity is approximately 100.0, the average intensity across all peaks for a sample is approximately 50.0. To ensure that no sample varied significantly from this average, each samples spectrum is scaled so that the sum of all peak intensities is exactly 15000.0.

Each dataset is constructed to contain the same number of Cases and Controls (30, 42, 60, 90, 150, and 300 Cases and Controls). For each number of Cases and Controls, a total of five random datasets are constructed, producing 30 unique datasets. For each spectrum in each dataset, peak 64 should have one of the lowest intensities, but it is possible to have a lower intensity in another peak, even peak 131, since the intensity is set to a random value between zero and the maximum allowed.

As described previously [14], the DT and MCA algorithms use a modified Evolutionary Programming (mEP) algorithm [32] to search for efficient classifiers. The DT algorithm is based on a symmetric seven-node decision tree (Figure 1). A classifier is represented by an array containing the peak numbers for the decision nodes 1 through 7, and an associated array of seven cut points used to determine which samples are assigned to each of the two daughter nodes. The mEP procedure ensures that each decision tree is unique in that the array of seven peak numbers is different for each tree and that no peak is used more than once. This means that two decision trees can use the same set of peaks, but their ordering must be different. For each array of peak numbers, the algorithm searches through a subset of putative cut points patterns and keeps the set of cut points that yield the highest quality. Therefore, the best decision tree obtained at the end of the run may not be optimum in that a change in one of more of the cut points may yield better results. In addition, a very good decision tree may not be found because either the mEP search did not explore this tree, or a set of sub-optimum cut points were located yielding a poorer quality.

Each time a decision tree is constructed, it is examined for possible pruning. Each decision node is examined before dividing the samples among the daughter nodes and if the number of Cases or Controls is less than a given fraction, *F*, of the total it is converted to a terminal node. Therefore, if Node 7 in Figure 1 has a small enough number of Cases or Controls, it becomes a terminal node. Daughter nodes 14 and 15 are removed and this decision tree only uses six features. If this decision tree is used to construct a new tree, the mEP procedure only allows for Nodes 1 through 6 to be changed so that this offspring only contains six peaks. This DT algorithm is run four times with different seeds to the random number generator for each dataset. Two of the runs set *F *to 1.0%. The other two runs set *F *to 4.0% so that a decision node is converted to a terminal node if the number of Cases or Controls is at most 1, 1, 2, 3, 6 or 12 for the datasets with 30, 42, 60, 90, 150, or 300 Cases and Controls, respectively. The terminal nodes of each decision tree are examined and either labeled as a Case Node, a Control Node, or an Undetermined Node. The samples in each node are then used to construct a 2 × 2 contingency table. For example, if a terminal node contains seven Cases and one Control, it is a Case Node and the number of true positives (NTP) is increased by seven and the number of false positives (NFP) is increased by one. If a terminal node contains two Cases and six Controls, it is denoted a Control Node and the number of false negatives (NFN) is increased by two and the number of true negatives (NTN) is increased by six. If the terminal node contains the same number of Cases and Controls, it is denoted an Undetermined Node and the classification of all samples in this node are one-half right and one-half wrong. Therefore, if this node contains three Cases and three Controls, NTP, NFP, NFN and NTN are all increased by 1.5. The quality of this decision tree is the sum of the sensitivity, NTP/(NTP+NFP), and the specificity, NTN/(NTN+NFN).

The mEP procedure uses a population size of 200 and runs for 400 generations if the dataset contains at most 90 Cases and 90 Controls, and a population size of 400 run for 800 generations if the dataset contains 150 or 300 Cases and Controls. Each time an offspring is created, any new features placed into the decision tree have 10 random cut points examined while keeping all other cut points fixed. All decision nodes then have their cut points set to five new random values and all combinations of cut points are examined to find the best combination of cut points for this set of features. This search is definitely not exhaustive, so the results should be taken as a lower bound to the accuracy that can be obtained from a dataset with no biological information.

The MCA algorithm is similar to the classification algorithm used in studies from the laboratories of Petricoin and Liotta [5-10] with some exceptions. Each run is assigned a fixed number of peaks, *N*, and the mEP procedure ensures that all sets of *N *peaks stored in the program are unique. The intensities of these peaks are linearly scaled to vary between 0.0 and 1.0 and a sample represents a new cell, or fingerprint pattern, if its Euclidean distance to any other examined sample is more than the trust radius of 0.1(N)^1/2^. The sample's category determines the category of this cell [5-10]. If the first sample is a Case, then it defines a Case Cell, or Case Fingerprint Pattern, and NTP is increased by one. If another Case sample lies within its trust radius, NTP is increased by one, while if a Control sample is within this trust radius, NFP is increased by one. Similarly if a Control sample has a sufficiently unique fingerprint it becomes the medoid of a Control Cell and NTN is increased by one. If a Case or Control sample has a fingerprint pattern that is within its trust radius, NFN or NTN is increased by one, respectively.

To ensure that this fingerprint pattern has complete coverage and can be divided into an effective training and testing set, the number of Case Cells or Control Cells cannot be more than 2/3 of the number of Cases or Controls. If this is true for a given panel of markers the quality is again the sum of the sensitivity and specificity, while if too many of either category of cell is produced the quality is set to zero. Three pairs of MCA runs are performed for each dataset, with the number of peaks used in the classifier (*N*) set to five, six or seven. For each value of *N*, the program is run by first examining all Cases and then all Controls, and by examining all Controls and then all Cases. The mEP procedure uses a population size of 400 and runs for 800 generations for datasets with 30, 42, 60 and 90 Cases and Controls. For the datasets with 150 and 300 Cases and Controls the population size is increased to 1000 and the search is run for 2000 generations. This larger search is also used to produce the results in Tables 2 and 3.

## Authors' contributions

BTL and JRC jointly devised this study and wrote the manuscript. BTL created the artificial datasets, wrote the software for the fingerprint-based classifiers, and performed the analysis.

## Supplementary Material

Additional file 1 **Decision tree classification accuracy for each dataset**. This table lists the classification accuracy (sum of the sensitivity and specificity as percentages) using the decision tree algorithm for the 1^st ^and 200^th ^best classifier as a function of the number of Cases and Controls. Click here for file

Additional file 2 **Medoid classifier algorithm accuracy for each dataset**. This table lists the classification accuracy (sum of the sensitivity and specificity as percentages) using the medoid classification algorithm for the 1st and 200th best classifier as a function of the number of Cases and Controls from two runs using five, six, and seven peaks with random intensities. Click here for file
