# Supplementary material for: Examining the significance of fingerprint-based classifiers
Source: BMC Bioinformatics. 2008 Dec 17;9:545. doi: 10.1186/1471-2105-9-545 (PMC2628908; doi:10.1186/1471-2105-9-545)
Supplement: Additional file 1 — Decision tree classification accuracy for each dataset. This table lists the classification accuracy (sum of the sensitivity and specificity as percentages) using the decision tree algorithm for the 1st and 200th best classifier as a function of the number of Cases and Controls. [file 1471-2105-9-545-S1.doc]

Additional file 1: Classification accuracy (sum of the sensitivity and specificity as percentages) using the decision tree algorithm for the 1^st^ and 200^th^ best classifier as a function of the number of Cases and Controls.^(a)^

| **Set** | **Cases** | **Controls** | **0%** | | **0%** | | **4%** | | **4%** | |
| --- | --- | --- | --- | --- | --- | --- | --- | --- | --- | --- |
|  |  |  | **1^st^** | **200^th^** | **1^st^** | **200^th^** | **1^st^** | **200^th^** | **1^st^** | **200^th^** |
| **30_1a** | **30** | **30** | 200.0 | 196.7 | 196.7 | 196.7 | 190.0 | 190.0 | 196.7 | 190.0 |
| **30_2a** | **30** | **30** | 196.7 | 196.7 | 196.7 | 193.3 | 196.7 | 193.3 | 200.0 | 196.7 |
| **30_3a** | **30** | **30** | 190.0 | 190.0 | 193.3 | 190.0 | 196.7 | 193.3 | 196.7 | 193.3 |
| **30_4a** | **30** | **30** | 196.7 | 196.7 | 196.7 | 193.3 | 193.3 | 186.7 | 193.3 | 193.3 |
| **30_5a** | **30** | **30** | 196.7 | 193.3 | 200.0 | 200.0 | 190.0 | 183.3 | 196.7 | 193.3 |
| **42_1a** | **42** | **42** | 188.1 | 185.7 | 185.8 | 178.5 | 183.3 | 180.9 | 183.3 | 180.9 |
| **42_2a** | **42** | **42** | 188.1 | 185.7 | 185.8 | 178.5 | 183.3 | 180.9 | 183.3 | 180.0 |
| **42_3a** | **42** | **42** | 183.3 | 183.3 | 188.1 | 185.7 | 185.7 | 185.7 | 183.3 | 180.9 |
| **42_4a** | **42** | **42** | 183.3 | 180.9 | 178.5 | 178.6 | 178.5 | 176.2 | 180.9 | 178.5 |
| **42_5a** | **42** | **42** | 190.5 | 185.7 | 185.7 | 180.9 | 188.1 | 185.7 | 180.9 | 176.2 |
| **60_1a** | **60** | **60** | 176.6 | 175.0 | 175.0 | 170.0 | 175.0 | 166.6 | 176.6 | 171.6 |
| **60_2a** | **60** | **60** | 176.6 | 171.6 | 175.0 | 171.6 | 171.6 | 168.3 | 173.3 | 168.3 |
| **60_3a** | **60** | **60** | 171.6 | 171.6 | 178.3 | 175.0 | 176.6 | 173.4 | 163.3 | 171.7 |
| **60_4a** | **60** | **60** | 176.6 | 173.3 | 176.6 | 171.6 | 176.7 | 173.3 | 171.6 | 168.3 |
| **60_5a** | **60** | **60** | 171.6 | 170.0 | 170.0 | 168.3 | 171.6 | 168.3 | 170.0 | 165.0 |
| **90_1a** | **90** | **90** | 156.7 | 155.6 | 160.0 | 158.9 | 160.0 | 158.9 | 162.3 | 158.9 |
| **90_2a** | **90** | **90** | 166.7 | 165.6 | 163.4 | 160.0 | 160.0 | 156.7 | 158.9 | 155.6 |
| **90_3a** | **90** | **90** | 158.9 | 157.8 | 161.1 | 158.9 | 158.9 | 156.7 | 164.5 | 152.2 |
| **90_4a** | **90** | **90** | 160.0 | 158.9 | 162.2 | 160.0 | 160.0 | 156.7 | 160.0 | 157.8 |
| **90_5a** | **90** | **90** | 166.7 | 164.4 | 162.2 | 158.9 | 166.7 | 165.6 | 161.1 | 158.9 |
| **150_1a** | **150** | **150** | 152.0 | 150.0 | 152.0 | 150.0 | 150.0 | 148.0 | 152.7 | 150.0 |
| **150_2a** | **150** | **150** | 149.3 | 146.0 | 150.7 | 149.3 | 150.7 | 149.3 | 151.3 | 150.0 |
| **150_3a** | **150** | **150** | 151.3 | 148.7 | 150.7 | 148.7 | 149.3 | 147.3 | 150.7 | 149.3 |
| **150_4a** | **150** | **150** | 152.0 | 150.0 | 149.3 | 148.0 | 155.3 | 154.0 | 153.3 | 151.3 |
| **150_5a** | **150** | **150** | 152.7 | 151.3 | 154.0 | 152.7 | 149.3 | 146.7 | 148.7 | 146.7 |
| **300_1a** | **300** | **300** | 138.3 | 137.3 | 136.3 | 135.3 | 137.3 | 136.0 | 135.7 | 134.7 |
| **300_2a** | **300** | **300** | 137.0 | 136.0 | 136.3 | 135.3 | 137.0 | 135.7 | 137.7 | 136.3 |
| **300_3a** | **300** | **300** | 136.7 | 136.0 | 137.0 | 136.0 | 134.0 | 132.0 | 136.3 | 135.0 |
| **300_4a** | **300** | **300** | 136.0 | 135.0 | 136.7 | 135.3 | 136.3 | 133.7 | 136.0 | 134.7 |
| **300_5a** | **300** | **300** | 135.0 | 133.7 | 136.3 | 135.0 | 135.7 | 134.7 | 135.7 | 135.0 |

^(a)^A decision node was converted to a terminal node if either it contained only samples from one of the categories for datasets with 90 Cases and Controls or less and 1% of the samples from either category for datasets with 150 and 300 Cases and Controls (0-1% runs) or if it contained less than 4% of the samples from either category (4% runs). All four runs used different seeds to the random number generator that controlled the Evolutionary Programming search.
