# Supplementary material for: Examining the significance of fingerprint-based classifiers
Source: BMC Bioinformatics. 2008 Dec 17;9:545. doi: 10.1186/1471-2105-9-545 (PMC2628908; doi:10.1186/1471-2105-9-545)
Supplement: Additional file 2 — Medoid classifier algorithm accuracy for each dataset. This table lists the classification accuracy (sum of the sensitivity and specificity as percentages) using the medoid classification algorithm for the 1st and 200th best classifier as a function of the number of Cases and Controls from two runs using five, six, and seven peaks with random intensities. [file 1471-2105-9-545-S2.doc]

Additional file 2: Classification accuracy (sum of the sensitivity and specificity as percentages) using the medoid classification algorithm for the 1st and 200th best classifier as a function of the number of Cases and Controls from two runs using five, six, and seven peaks with random intensities.^(a)^

| **Set** | **Cases** | **Controls** | **Run** | **5 Peaks** | | **6 Peaks** | | **7 Peaks** | |
| --- | --- | --- | --- | --- | --- | --- | --- | --- | --- |
|  |  |  |  | **1^st^** | **200^th^** | **1^st^** | **200^th^** | **1^st^** | **200^th^** |
| **30_1a** | **30** | **30** | **1** | 200.0 | 193.3 | 200.0 | 196.7 | 200.0 | 196.7 |
|  |  |  | **2** | 200.0 | 193.3 | 200.0 | 196.7 | 200.0 | 200.0 |
| **30_2a** | **30** | **30** | **1** | 200.0 | 193.3 | 200.0 | 196.7 | 200.0 | 196.7 |
|  |  |  | **2** | 200.0 | 193.3 | 200.0 | 196.7 | 200.0 | 196.7 |
| **30_3a** | **30** | **30** | **1** | 200.0 | 190.0 | 200.0 | 196.7 | 200.0 | 200.0 |
|  |  |  | **2** | 200.0 | 193.3 | 200.0 | 200.0 | 200.0 | 196.7 |
| **30_4a** | **30** | **30** | **1** | 200.0 | 193.3 | 200.0 | 196.7 | 200.0 | 196.7 |
|  |  |  | **2** | 200.0 | 193.3 | 200.0 | 193.3 | 200.0 | 200.0 |
| **30_5a** | **30** | **30** | **1** | 200.0 | 193.3 | 200.0 | 200.0 | 200.0 | 196.7 |
|  |  |  | **2** | 200.0 | 193.3 | 200.0 | 196.7 | 200.0 | 200.0 |
| **42_1a** | **42** | **42** | **1** | 195.2 | 188.1 | 197.6 | 190.5 | 197.6 | 192.9 |
|  |  |  | **2** | 195.2 | 188.1 | 197.6 | 190.5 | 197.6 | 192.9 |
| **42_2a** | **42** | **42** | **1** | 197.6 | 188.1 | 197.6 | 192.9 | 197.6 | 192.9 |
|  |  |  | **2** | 192.9 | 188.1 | 197.6 | 192.9 | 197.6 | 195.2 |
| **42_3a** | **42** | **42** | **1** | 192.9 | 188.1 | 197.6 | 190.5 | 197.6 | 192.9 |
|  |  |  | **2** | 195.2 | 188.1 | 195.2 | 190.5 | 197.6 | 192.9 |
| **42_4a** | **42** | **42** | **1** | 195.2 | 188.1 | 197.6 | 190.5 | 197.6 | 192.9 |
|  |  |  | **2** | 195.2 | 188.1 | 195.2 | 190.5 | 197.6 | 195.2 |
| **42_5a** | **42** | **42** | **1** | 192.9 | 185.7 | 195.2 | 188.1 | 197.6 | 192.9 |
|  |  |  | **2** | 195.2 | 188.1 | 197.6 | 192.9 | 197.6 | 195.2 |
| **60_1a** | **60** | **60** | **1** | 191.7 | 183.3 | 191.7 | 185.0 | 195.0 | 190.0 |
|  |  |  | **2** | 188.3 | 181.7 | 193.3 | 186.7 | 195.0 | 190.0 |
| **60_2a** | **60** | **60** | **1** | 188.3 | 181.7 | 193.3 | 185.0 | 195.0 | 186.7 |
|  |  |  | **2** | 193.3 | 183.3 | 193.3 | 185.0 | 193.3 | 186.7 |
| **60_3a** | **60** | **60** | **1** | 190.0 | 183.3 | 192.7 | 185.0 | 193.3 | 186.7 |
|  |  |  | **2** | 193.3 | 183.3 | 193.3 | 186.7 | 193.3 | 188.3 |
| **60_4a** | **60** | **60** | **1** | 193.3 | 183.3 | 193.3 | 185.0 | 195.0 | 190.0 |
|  |  |  | **2** | 190.0 | 183.3 | 193.3 | 185.0 | 191.7 | 186.7 |
| **60_5a** | **60** | **60** | **1** | 193.3 | 181.7 | 193.3 | 186.7 | 195.0 | 190.0 |
|  |  |  | **2** | 190.0 | 183.3 | 191.7 | 186.7 | 193.3 | 190.0 |
| **90_1a** | **90** | **90** | **1** | 184.4 | 178.9 | 188.9 | 181.1 | 188.9 | 183.3 |
|  |  |  | **2** | 184.4 | 177.8 | 185.6 | 180.0 | 188.9 | 182.2 |
| **90_2a** | **90** | **90** | **1** | 185.6 | 177.8 | 188.9 | 181.1 | 190.0 | 184.4 |
|  |  |  | **2** | 184.4 | 178.9 | 186.7 | 180.0 | 190.0 | 183.3 |
| **90_3a** | **90** | **90** | **1** | 184.4 | 177.8 | 186.7 | 180.0 | 191.1 | 183.3 |
|  |  |  | **2** | 187.8 | 178.9 | 188.9 | 182.2 | 188.9 | 184.4 |
| **90_4a** | **90** | **90** | **1** | 183.3 | 178.9 | 186.7 | 181.1 | 188.9 | 182.2 |
|  |  |  | **2** | 185.6 | 177.8 | 187.8 | 181.1 | 190.0 | 182.2 |
| **90_5a** | **90** | **90** | **1** | 185.6 | 177.8 | 185.6 | 180.0 | 188.9 | 182.2 |
|  |  |  | **2** | 186.7 | 181.1 | 187.8 | 182.2 | 190.0 | 185.6 |
| **150_1a** | **150** | **150** | **1** | 182.0 | 176.0 | 183.3 | 179.3 | 186.7 | 181.3 |
|  |  |  | **2** | 180.0 | 174.0 | 182.0 | 178.0 | 184.7 | 179.3 |
| **150_2a** | **150** | **150** | **1** | 183.3 | 174.7 | 182.7 | 178.7 | 184.7 | 180.7 |
|  |  |  | **2** | 181.3 | 174.7 | 182.7 | 178.0 | 187.3 | 180.0 |
| **150_3a** | **150** | **150** | **1** | 180.0 | 174.7 | 183.3 | 178.7 | 184.0 | 180.7 |
|  |  |  | **2** | 180.0 | 175.3 | 184.0 | 179.3 | 185.3 | 180.7 |
| **150_4a** | **150** | **150** | **1** | 180.7 | 175.3 | 182.0 | 178.7 | 184.0 | 180.7 |
|  |  |  | **2** | 180.0 | 174.7 | 185.3 | 178.7 | 185.3 | 181.3 |
| **150_5a** | **150** | **150** | **1** | 182.0 | 175.3 | 182.7 | 178.7 | 185.3 | 180.7 |
|  |  |  | **2** | 180.0 | 174.0 | 182.7 | 178.0 | 184.0 | 180.0 |
| **300_1a** | **300** | **300** | **1** | 167.3 | 163.3 | 176.3 | 171.7 | 179.7 | 175.0 |
|  |  |  | **2** | 170.3 | 163.3 | 175.7 | 172.3 | 178.7 | 175.0 |
| **300_2a** | **300** | **300** | **1** | 169.7 | 163.3 | 175.3 | 171.3 | 178.7 | 174.0 |
|  |  |  | **2** | 169.3 | 163.3 | 179.0 | 172.7 | 180.3 | 175.7 |
| **300_3a** | **300** | **300** | **1** | 168.0 | 163.3 | 175.3 | 172.3 | 179.3 | 175.3 |
|  |  |  | **2** | 168.7 | 163.3 | 178.3 | 172.3 | 178.7 | 175.3 |
| **300_4a** | **300** | **300** | **1** | 168.0 | 163.3 | 176.7 | 172.3 | 178.3 | 175.3 |
|  |  |  | **2** | 167.0 | 163.3 | 176.7 | 171.3 | 178.7 | 175.0 |
| **300_5a** | **300** | **300** | **1** | 168.3 | 163.3 | 176.7 | 171.7 | 178.7 | 174.3 |
|  |  |  | **2** | 168.3 | 163.7 | 176.3 | 172.7 | 178.7 | 175.0 |

^(a)^Run 1 examined all Cases and then Controls, while Run 2 examined the Controls and then the Cases.
